# *catena*-Poly[[bis­[1-phenyl-3-(1*H*-1,2,4-triazol-1-yl)propan-1-one-κ*N* ^4^]cadmium(II)]-di-μ-azido-κ^4^ *N* ^1^:*N* ^3^]

**DOI:** 10.1107/S1600536808012609

**Published:** 2008-05-07

**Authors:** Ying Guo, Hua Cai, Jiangang Li, Guobao Wan

**Affiliations:** a College of Science, Civil Aviation University of China, Tianjin 300300, People’s Republic of China

## Abstract

In the crystal structure of the title complex, [Cd(N_3_)_2_(C_11_H_11_N_31_)_2_]_*n*_, there are two crystallographically independent Cd^II^ atoms. Both exist in an octa­hedral environment composed of four N atoms of the N_3_^−^ groups and two N atoms from two monodentate 1-phenyl-3-(1*H*-1,2,4-triazol-1-yl)propan-1-one ligands that are positioned *trans* to each other. Adjacent Cd^II^ centres in the crystal structure are bridged by a pair of N_3_^−^ anions in a μ-1,3-fashion, forming an infinite one-dimensional array.

## Related literature

For related literature, see: Guo & Cai (2007 ▶).
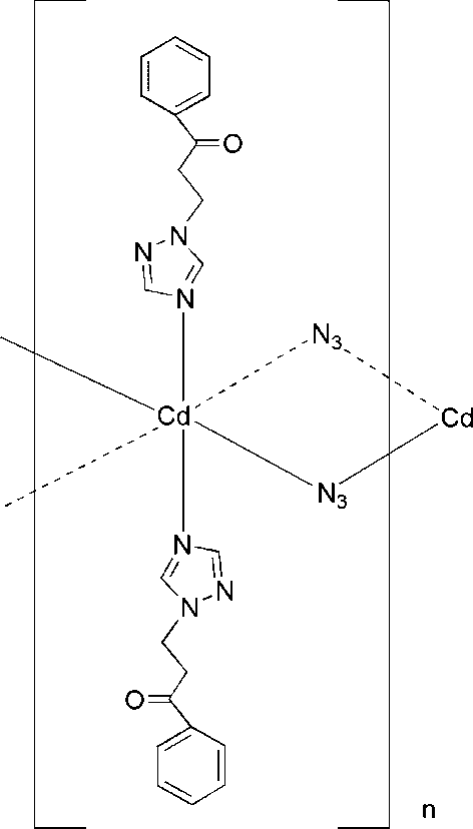


## Experimental

### 

#### Crystal data

[Cd(N_3_)_2_(C_11_H_11_N_31_)_2_]
*M* *_r_* = 598.92
Monoclinic, 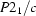

*a* = 10.1626 (16) Å
*b* = 17.732 (3) Å
*c* = 28.124 (4) Å
β = 91.980 (2)°
*V* = 5064.9 (14) Å^3^
*Z* = 8
Mo *K*α radiation
μ = 0.91 mm^−1^
*T* = 294 (2) K
0.24 × 0.22 × 0.14 mm

#### Data collection

Bruker SMART CCD area-detector diffractometer
Absorption correction: multi-scan (*SADABS*; Sheldrick, 2004 ▶) *T*_min_ = 0.760, *T*_max_ = 1.000 (expected range = 0.670–0.881)
26797 measured reflections
8928 independent reflections
7054 reflections with *I* > 2σ(*I*)
*R*_int_ = 0.021

#### Refinement

*R*[*F* ^2^ > 2σ(*F* ^2^)] = 0.031
*wR*(*F* ^2^) = 0.069
*S* = 1.09
8928 reflections
667 parameters
H-atom parameters constrained
Δρ_max_ = 0.67 e Å^−3^
Δρ_min_ = −0.72 e Å^−3^

### 

Data collection: *SMART* (Bruker, 2001 ▶); cell refinement: *SAINT* (Bruker, 2001 ▶); data reduction: *SAINT*; program(s) used to solve structure: *SHELXS97* (Sheldrick, 2008 ▶); program(s) used to refine structure: *SHELXL97* (Sheldrick, 2008 ▶); molecular graphics: *SHELXTL* (Sheldrick, 2008 ▶); software used to prepare material for publication: *SHELXTL*.

## Supplementary Material

Crystal structure: contains datablocks I, global. DOI: 10.1107/S1600536808012609/bv2096sup1.cif

Structure factors: contains datablocks I. DOI: 10.1107/S1600536808012609/bv2096Isup2.hkl

Additional supplementary materials:  crystallographic information; 3D view; checkCIF report

## Figures and Tables

**Fig. 1.**
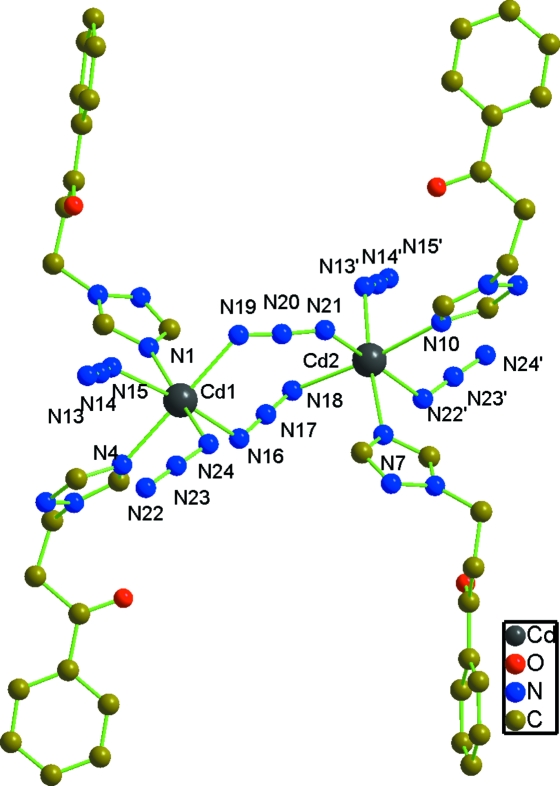
The molecular structure of (I), with atom labels and 30% probability displacement ellipsoids.

**Fig. 2.**
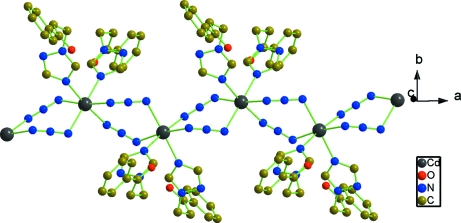
One-dimensional chain connected by N3-. H atoms have been omitted.

## supplementary crystallographic information

### Comment

Pseudohalide anions N_3_^-^, NCS^-^ and NCO^-^ are known as extremely versatile ligands in coordination chemistry because of their multiple bridging modes. Recently, we initiated a research program involving synthesizing supramolecules based on a pseudohalide and a flexible ligand consisting of a propanone unit substituted with an imidazole and a phenyl group (Cai & Guo, 2007). To further explore this series, we synthesized the title compound, (I), a new Cd^II^ complex based on the mixed ligands azide and 3-(1*H*-1,2,4-triazol-1-yl) -1-phenylpropan-1-one (*L*), which consists of a propanone unit substituted with an triazole and a phenyl group.

The X-ray crystallographic analysis shows that the structure of complex I consists of 1-D Cd^II^ chains with two Cd^II^ atoms in the asymmetric unit. Two crystallographically independent Cd^II^ ions have similar coordination environment, but different bond parameters. In both cases, each Cd^II^ atom is in a distorted N_6_ octahedron environment, consisting of two terminal and two bridging azide N atoms and N atoms from two ligand (*L*) (arranged in a *cis* fation). The Cd—N bond distance range from 2.313 (3)Å to 2.369 (3) Å. Cd1 and Cd2 are displaced by 0.2047 and 0.7313Å from the mean basal plane toward the apical nitrogen atoms (N24 and N7). In this structure, the ligand *L* displays monodentate binding to Cd^II^ and the triazole and phenyl rings in each of the ligands are not coplanar. The dihedral angels formed by the least-squares planes of the phenyl and triazole rings are 78.1 (1)°, 74.5 (1)° 74.3 (1)° and 77.5 (1)°, respectively. Adjacent Cd^II^ centers are bridged by two azide anions in an end-to-end (µ-1,3) fashion forming a 1-D chain polymer, as illustrated in Fig.2. The minimum interdinuclear Cd···Cd distance is 5.415 Å.

### Experimental

All the starting materials and solvents for syntheses were obtained commercially and used as received. Cd(NO_3_)_2_.4H_2_O (30.8 mg, 0.1 mmol), 3-(1*H*-1,2,4-triazol-1-yl)-1-phenylpropan-1-one (22.3 mg, 0.1 mmol) and NaN_3_ (6.7 mg, 0.1 mmol) were mixed in a CH_3_CN–H_2_O (20 ml, 1:1 *v*/*v*) solution with vigorous stirring for *ca* 30 min. The resulting solution was filtered and left to stand at room temperature. Colourless prismatic crystals of I suitable for X-ray analysis were obtained in 60% yield by slow evaporation of the solvent over a period of 1 week. Analysis, calculated for CdC_22_H_22_N_12_O_2_: C 44.12, H 3.70, N 28.06; found: C 44.15, H 3.83, N 28.10.

### Refinement

Although all H atoms were visible in difference maps, they were placed in geometrically calculated positions, with C—H distances in the range 0.93–0.97 Å, and included in the final refinement in the riding model approximation,with *U*_iso_(H) = 1.2*U*_eq_(C) for aromatic and methylene H atoms.

### Crystal data

**Table:**

[Cd(N_3_)_2_(C_11_H_11_N_3_)_2_]	*F*_000_ = 2416
*M**_r_* = 598.92	*D*_x_ = 1.571 Mg m^−^^3^
Monoclinic, *P*2_1_/*c*	Mo *K*α radiation λ = 0.71073 Å
Hall symbol: -P 2ybc	Cell parameters from 8981 reflections
*a* = 10.1626 (16) Å	θ = 2.4–27.9º
*b* = 17.732 (3) Å	µ = 0.91 mm^−^^1^
*c* = 28.124 (4) Å	*T* = 294 (2) K
β = 91.980 (2)º	Block, pale yellow
*V* = 5064.9 (14) Å^3^	0.24 × 0.22 × 0.14 mm
*Z* = 8	

### Data collection

**Table:**

Bruker SMART CCD area-detector diffractometer	8928 independent reflections
Radiation source: fine-focus sealed tube	7054 reflections with *I* > 2σ(*I*)
Monochromator: graphite	*R*_int_ = 0.021
*T* = 294(2) K	θ_max_ = 25.0º
φ and ω scans	θ_min_ = 1.9º
Absorption correction: multi-scan(SADABS; Sheldrick, 2004)	*h* = −12→12
*T*_min_ = 0.760, *T*_max_ = 1.000	*k* = −21→17
26797 measured reflections	*l* = −30→33

### Refinement

**Table:**

Refinement on *F*^2^	Secondary atom site location: difference Fourier map
Least-squares matrix: full	Hydrogen site location: inferred from neighbouring sites
*R*[*F*^2^ > 2σ(*F*^2^)] = 0.031	H-atom parameters constrained
*wR*(*F*^2^) = 0.069	*w* = 1/[σ^2^(*F*_o_^2^) + (0.0145*P*)^2^ + 5.5025*P*] where *P* = (*F*_o_^2^ + 2*F*_c_^2^)/3
*S* = 1.09	(Δ/σ)_max_ = 0.001
8928 reflections	Δρ_max_ = 0.67 e Å^−^^3^
667 parameters	Δρ_min_ = −0.72 e Å^−^^3^
Primary atom site location: structure-invariant direct methods	Extinction correction: none

### Special details

**Table:**

Geometry. All e.s.d.'s (except the e.s.d. in the dihedral angle between two l.s. planes) are estimated using the full covariance matrix. The cell e.s.d.'s are taken into account individually in the estimation of e.s.d.'s in distances, angles and torsion angles; correlations between e.s.d.'s in cell parameters are only used when they are defined by crystal symmetry. An approximate (isotropic) treatment of cell e.s.d.'s is used for estimating e.s.d.'s involving l.s. planes.
Refinement. Refinement of *F*^2^ against ALL reflections. The weighted *R*-factor *wR* and goodness of fit *S* are based on *F*^2^, conventional *R*-factors *R* are based on *F*, with *F* set to zero for negative *F*^2^. The threshold expression of *F*^2^ > σ(*F*^2^) is used only for calculating *R*-factors(gt) *etc*. and is not relevant to the choice of reflections for refinement. *R*-factors based on *F*^2^ are statistically about twice as large as those based on *F*, and *R*- factors based on ALL data will be even larger.

### Fractional atomic coordinates and isotropic or equivalent isotropic displacement parameters (Å^2^)

**Table:**

	*x*	*y*	*z*	*U*_iso_*/*U*_eq_	
Cd1	0.50852 (2)	0.061464 (13)	0.240599 (8)	0.03961 (7)	
Cd2	1.00703 (2)	0.168651 (13)	0.237148 (8)	0.03910 (7)	
O1	0.4692 (3)	−0.11356 (17)	0.06491 (9)	0.0803 (9)	
O2	0.5268 (3)	−0.09785 (16)	0.44068 (9)	0.0737 (8)	
O3	0.9832 (3)	0.34977 (16)	0.05865 (9)	0.0758 (8)	
O4	1.0136 (3)	0.33667 (16)	0.43755 (10)	0.0801 (9)	
N1	0.5899 (2)	−0.04669 (16)	0.20487 (9)	0.0472 (7)	
N2	0.7191 (3)	−0.13452 (19)	0.17430 (11)	0.0653 (9)	
N3	0.5917 (2)	−0.14797 (16)	0.16199 (9)	0.0437 (7)	
N4	0.4138 (2)	−0.01421 (16)	0.29893 (9)	0.0473 (7)	
N5	0.2778 (3)	−0.09947 (19)	0.32893 (11)	0.0618 (8)	
N6	0.4052 (2)	−0.11606 (16)	0.34126 (9)	0.0457 (7)	
N7	0.9118 (2)	0.24449 (17)	0.29467 (9)	0.0484 (7)	
N8	0.7799 (3)	0.3330 (2)	0.32370 (11)	0.0651 (9)	
N9	0.9081 (2)	0.34863 (16)	0.33551 (9)	0.0453 (7)	
N10	1.0898 (2)	0.27623 (16)	0.20045 (9)	0.0454 (7)	
N11	1.2206 (3)	0.36462 (19)	0.17075 (11)	0.0641 (9)	
N12	1.0927 (2)	0.37879 (15)	0.15837 (9)	0.0425 (6)	
N13	0.1203 (3)	0.10059 (19)	0.18182 (10)	0.0608 (8)	
N14	0.2190 (3)	0.06887 (15)	0.18986 (8)	0.0409 (6)	
N15	0.3163 (3)	0.03539 (18)	0.19487 (10)	0.0578 (8)	
N16	0.6970 (3)	0.06784 (18)	0.29227 (9)	0.0570 (8)	
N17	0.8037 (3)	0.06395 (15)	0.27865 (9)	0.0462 (7)	
N18	0.9121 (3)	0.05811 (17)	0.26561 (11)	0.0607 (8)	
N19	0.6188 (3)	0.1277 (2)	0.18341 (10)	0.0698 (10)	
N20	0.7180 (3)	0.15975 (16)	0.18864 (8)	0.0431 (6)	
N21	0.8162 (3)	0.19275 (18)	0.19088 (10)	0.0602 (8)	
N22	1.1938 (3)	0.16446 (18)	0.28984 (10)	0.0562 (8)	
N23	1.3020 (3)	0.16754 (15)	0.27785 (9)	0.0457 (7)	
N24	1.4122 (3)	0.17232 (17)	0.26625 (12)	0.0642 (9)	
C1	0.5177 (3)	−0.0957 (2)	0.18030 (11)	0.0475 (8)	
H1	0.4265	−0.0936	0.1765	0.057*	
C2	0.7122 (3)	−0.0734 (2)	0.20004 (13)	0.0623 (11)	
H2	0.7862	−0.0503	0.2139	0.075*	
C3	0.5523 (4)	−0.2140 (2)	0.13389 (11)	0.0533 (9)	
H3A	0.4571	−0.2150	0.1302	0.064*	
H3B	0.5797	−0.2592	0.1510	0.064*	
C4	0.6107 (3)	−0.21481 (19)	0.08523 (10)	0.0503 (8)	
H4A	0.7045	−0.2052	0.0887	0.060*	
H4B	0.5993	−0.2647	0.0716	0.060*	
C5	0.5504 (3)	−0.1578 (2)	0.05133 (11)	0.0491 (8)	
C6	0.5919 (3)	−0.1582 (2)	0.00092 (11)	0.0496 (8)	
C7	0.6808 (4)	−0.2096 (2)	−0.01580 (13)	0.0677 (11)	
H7	0.7188	−0.2451	0.0048	0.081*	
C8	0.7136 (4)	−0.2087 (3)	−0.06316 (15)	0.0869 (14)	
H8	0.7745	−0.2431	−0.0742	0.104*	
C9	0.6573 (5)	−0.1578 (3)	−0.09350 (15)	0.0893 (15)	
H9	0.6787	−0.1583	−0.1254	0.107*	
C10	0.5698 (5)	−0.1060 (3)	−0.07781 (15)	0.0910 (15)	
H10	0.5327	−0.0707	−0.0987	0.109*	
C11	0.5366 (4)	−0.1064 (2)	−0.03040 (13)	0.0729 (11)	
H11	0.4764	−0.0713	−0.0196	0.087*	
C12	0.2887 (3)	−0.0380 (2)	0.30351 (12)	0.0576 (10)	
H12	0.2165	−0.0130	0.2898	0.069*	
C13	0.4829 (3)	−0.06464 (19)	0.32326 (11)	0.0457 (8)	
H13	0.5742	−0.0641	0.3272	0.055*	
C14	0.4417 (4)	−0.1862 (2)	0.36616 (12)	0.0570 (9)	
H14A	0.5368	−0.1883	0.3703	0.068*	
H14B	0.4142	−0.2288	0.3465	0.068*	
C15	0.3809 (3)	−0.1935 (2)	0.41427 (11)	0.0540 (9)	
H15A	0.2876	−0.1824	0.4109	0.065*	
H15B	0.3899	−0.2453	0.4250	0.065*	
C16	0.4416 (3)	−0.1423 (2)	0.45150 (12)	0.0512 (8)	
C17	0.3961 (3)	−0.14650 (19)	0.50138 (12)	0.0480 (8)	
C18	0.4449 (4)	−0.0950 (2)	0.53441 (14)	0.0665 (10)	
H18	0.5055	−0.0589	0.5253	0.080*	
C19	0.4047 (5)	−0.0965 (3)	0.58076 (15)	0.0816 (13)	
H19	0.4376	−0.0614	0.6027	0.098*	
C20	0.3166 (5)	−0.1496 (3)	0.59435 (15)	0.0784 (13)	
H20	0.2890	−0.1504	0.6255	0.094*	
C21	0.2687 (4)	−0.2015 (3)	0.56260 (15)	0.0798 (13)	
H21	0.2097	−0.2380	0.5724	0.096*	
C22	0.3071 (4)	−0.2003 (2)	0.51579 (13)	0.0648 (10)	
H22	0.2731	−0.2355	0.4941	0.078*	
C23	0.7883 (3)	0.2704 (2)	0.29933 (13)	0.0621 (10)	
H23	0.7148	0.2458	0.2863	0.075*	
C24	0.9835 (3)	0.29546 (19)	0.31817 (11)	0.0458 (8)	
H24	1.0748	0.2940	0.3220	0.055*	
C25	0.9481 (4)	0.4190 (2)	0.35863 (12)	0.0566 (9)	
H25A	0.9290	0.4604	0.3369	0.068*	
H25B	1.0426	0.4179	0.3647	0.068*	
C26	0.8818 (3)	0.4346 (2)	0.40490 (12)	0.0550 (9)	
H26A	0.8928	0.4874	0.4130	0.066*	
H26B	0.7882	0.4248	0.4007	0.066*	
C27	0.9364 (3)	0.3870 (2)	0.44546 (13)	0.0527 (9)	
C28	0.8959 (3)	0.4038 (2)	0.49479 (12)	0.0526 (9)	
C29	0.8199 (4)	0.4657 (2)	0.50574 (14)	0.0670 (11)	
H29	0.7900	0.4982	0.4818	0.080*	
C30	0.7885 (4)	0.4793 (3)	0.55244 (16)	0.0836 (13)	
H30	0.7377	0.5211	0.5597	0.100*	
C31	0.8313 (5)	0.4320 (3)	0.58799 (16)	0.0857 (14)	
H31	0.8091	0.4413	0.6192	0.103*	
C32	0.9068 (5)	0.3708 (3)	0.57762 (15)	0.0902 (15)	
H32	0.9365	0.3387	0.6018	0.108*	
C33	0.9391 (4)	0.3566 (2)	0.53122 (14)	0.0730 (12)	
H33	0.9905	0.3149	0.5244	0.088*	
C34	1.0186 (3)	0.3258 (2)	0.17610 (11)	0.0479 (8)	
H34	0.9275	0.3236	0.1720	0.057*	
C35	1.2127 (3)	0.3027 (2)	0.19569 (12)	0.0596 (10)	
H35	1.2864	0.2787	0.2091	0.072*	
C36	1.0534 (3)	0.44613 (19)	0.13156 (11)	0.0514 (8)	
H36A	1.0768	0.4903	0.1503	0.062*	
H36B	0.9585	0.4459	0.1267	0.062*	
C37	1.1161 (3)	0.45241 (19)	0.08383 (11)	0.0489 (8)	
H37A	1.1033	0.5033	0.0718	0.059*	
H37B	1.2101	0.4441	0.0881	0.059*	
C38	1.0618 (3)	0.3976 (2)	0.04744 (11)	0.0472 (8)	
C39	1.1059 (3)	0.40312 (19)	−0.00248 (11)	0.0449 (8)	
C40	1.0632 (4)	0.3493 (2)	−0.03517 (13)	0.0629 (10)	
H40	1.0088	0.3104	−0.0257	0.076*	
C41	1.1009 (5)	0.3532 (2)	−0.08185 (14)	0.0760 (12)	
H41	1.0719	0.3168	−0.1036	0.091*	
C42	1.1803 (4)	0.4101 (3)	−0.09617 (14)	0.0738 (12)	
H42	1.2057	0.4122	−0.1276	0.089*	
C43	1.2228 (4)	0.4640 (3)	−0.06451 (14)	0.0747 (12)	
H43	1.2765	0.5029	−0.0745	0.090*	
C44	1.1860 (3)	0.4608 (2)	−0.01754 (12)	0.0596 (10)	
H44	1.2153	0.4975	0.0039	0.072*	

### Atomic displacement parameters (Å^2^)

**Table:**

	*U*^11^	*U*^22^	*U*^33^	*U*^12^	*U*^13^	*U*^23^
Cd1	0.02805 (11)	0.04518 (14)	0.04591 (13)	−0.00049 (10)	0.00570 (9)	−0.00056 (11)
Cd2	0.02820 (11)	0.04316 (14)	0.04624 (13)	−0.00053 (10)	0.00576 (9)	0.00231 (10)
O1	0.095 (2)	0.088 (2)	0.0581 (16)	0.0446 (18)	0.0029 (14)	−0.0031 (15)
O2	0.0821 (19)	0.0700 (19)	0.0689 (17)	−0.0344 (16)	0.0020 (14)	0.0044 (14)
O3	0.096 (2)	0.071 (2)	0.0602 (16)	−0.0429 (17)	0.0034 (14)	0.0020 (14)
O4	0.102 (2)	0.0661 (19)	0.0718 (18)	0.0399 (17)	−0.0006 (15)	−0.0043 (14)
N1	0.0327 (14)	0.0584 (19)	0.0509 (15)	0.0039 (13)	0.0053 (11)	−0.0117 (14)
N2	0.0376 (16)	0.082 (2)	0.075 (2)	0.0148 (15)	−0.0063 (14)	−0.0310 (19)
N3	0.0375 (14)	0.0548 (19)	0.0388 (14)	0.0008 (13)	0.0002 (11)	−0.0063 (13)
N4	0.0322 (14)	0.061 (2)	0.0493 (15)	−0.0042 (13)	0.0060 (12)	0.0060 (14)
N5	0.0357 (16)	0.076 (2)	0.073 (2)	−0.0120 (15)	0.0021 (14)	0.0167 (18)
N6	0.0381 (15)	0.0515 (19)	0.0476 (15)	−0.0049 (13)	0.0033 (12)	0.0012 (13)
N7	0.0337 (14)	0.061 (2)	0.0504 (16)	0.0009 (13)	0.0076 (12)	−0.0027 (14)
N8	0.0365 (16)	0.080 (2)	0.079 (2)	0.0155 (16)	0.0037 (14)	−0.0164 (19)
N9	0.0372 (15)	0.0497 (19)	0.0492 (15)	0.0091 (13)	0.0040 (12)	0.0007 (13)
N10	0.0333 (14)	0.0519 (18)	0.0511 (15)	−0.0004 (13)	0.0059 (11)	0.0112 (13)
N11	0.0367 (16)	0.082 (2)	0.073 (2)	−0.0132 (15)	−0.0052 (14)	0.0301 (18)
N12	0.0385 (14)	0.0466 (18)	0.0422 (14)	−0.0032 (13)	−0.0004 (11)	0.0036 (12)
N13	0.0482 (17)	0.076 (2)	0.0580 (18)	0.0179 (17)	−0.0023 (14)	−0.0157 (16)
N14	0.0380 (15)	0.0452 (17)	0.0394 (14)	−0.0024 (13)	0.0004 (11)	−0.0090 (12)
N15	0.0405 (16)	0.064 (2)	0.0684 (19)	0.0066 (15)	−0.0114 (14)	−0.0116 (16)
N16	0.0415 (17)	0.080 (2)	0.0490 (16)	−0.0104 (15)	0.0005 (13)	0.0002 (15)
N17	0.0451 (17)	0.0447 (17)	0.0484 (15)	−0.0092 (13)	−0.0067 (13)	0.0127 (13)
N18	0.0431 (17)	0.054 (2)	0.085 (2)	−0.0081 (15)	0.0063 (15)	0.0174 (16)
N19	0.0513 (18)	0.099 (3)	0.0590 (19)	−0.0305 (19)	−0.0032 (14)	0.0234 (18)
N20	0.0378 (15)	0.0520 (18)	0.0392 (14)	0.0007 (14)	−0.0014 (11)	0.0113 (12)
N21	0.0398 (16)	0.072 (2)	0.0675 (19)	−0.0107 (16)	−0.0099 (13)	0.0173 (17)
N22	0.0398 (16)	0.079 (2)	0.0502 (16)	0.0108 (15)	0.0001 (13)	−0.0016 (15)
N23	0.0460 (17)	0.0425 (17)	0.0478 (15)	0.0077 (13)	−0.0092 (13)	−0.0092 (13)
N24	0.0423 (18)	0.053 (2)	0.097 (2)	−0.0007 (15)	0.0045 (16)	−0.0172 (17)
C1	0.0293 (16)	0.060 (2)	0.0533 (19)	−0.0016 (16)	0.0060 (14)	−0.0069 (17)
C2	0.0345 (18)	0.082 (3)	0.070 (2)	0.0063 (18)	−0.0061 (16)	−0.030 (2)
C3	0.067 (2)	0.049 (2)	0.0432 (18)	−0.0044 (18)	−0.0014 (16)	−0.0032 (16)
C4	0.062 (2)	0.046 (2)	0.0429 (18)	0.0074 (17)	−0.0039 (15)	−0.0061 (15)
C5	0.054 (2)	0.046 (2)	0.0466 (19)	0.0050 (17)	−0.0017 (15)	−0.0034 (16)
C6	0.055 (2)	0.052 (2)	0.0420 (18)	−0.0061 (17)	−0.0038 (15)	−0.0009 (16)
C7	0.066 (2)	0.086 (3)	0.052 (2)	0.011 (2)	0.0080 (18)	0.000 (2)
C8	0.076 (3)	0.122 (4)	0.064 (3)	0.003 (3)	0.020 (2)	−0.010 (3)
C9	0.108 (4)	0.111 (4)	0.050 (2)	−0.031 (3)	0.016 (3)	0.006 (3)
C10	0.134 (4)	0.084 (4)	0.053 (3)	−0.010 (3)	−0.010 (3)	0.020 (2)
C11	0.097 (3)	0.065 (3)	0.057 (2)	0.009 (2)	−0.004 (2)	0.003 (2)
C12	0.0357 (18)	0.076 (3)	0.061 (2)	−0.0016 (18)	0.0047 (15)	0.017 (2)
C13	0.0318 (16)	0.056 (2)	0.0500 (18)	−0.0032 (16)	0.0049 (14)	0.0006 (17)
C14	0.066 (2)	0.046 (2)	0.059 (2)	−0.0013 (18)	0.0030 (17)	−0.0015 (18)
C15	0.061 (2)	0.044 (2)	0.057 (2)	−0.0091 (17)	−0.0012 (16)	0.0073 (17)
C16	0.048 (2)	0.046 (2)	0.059 (2)	−0.0029 (17)	−0.0041 (16)	0.0055 (17)
C17	0.0493 (19)	0.040 (2)	0.054 (2)	0.0044 (16)	−0.0054 (15)	0.0068 (16)
C18	0.088 (3)	0.044 (2)	0.067 (2)	−0.004 (2)	−0.007 (2)	0.001 (2)
C19	0.118 (4)	0.062 (3)	0.065 (3)	0.013 (3)	−0.008 (3)	−0.010 (2)
C20	0.090 (3)	0.088 (4)	0.058 (3)	0.026 (3)	0.009 (2)	0.009 (2)
C21	0.070 (3)	0.102 (4)	0.068 (3)	−0.012 (3)	0.009 (2)	0.017 (3)
C22	0.060 (2)	0.073 (3)	0.060 (2)	−0.014 (2)	−0.0021 (18)	0.008 (2)
C23	0.0306 (18)	0.086 (3)	0.070 (2)	0.0037 (18)	0.0050 (16)	−0.016 (2)
C24	0.0325 (16)	0.051 (2)	0.0541 (19)	0.0082 (16)	0.0075 (14)	0.0047 (17)
C25	0.057 (2)	0.049 (2)	0.064 (2)	0.0054 (18)	0.0049 (17)	0.0031 (18)
C26	0.057 (2)	0.046 (2)	0.062 (2)	0.0106 (17)	0.0042 (17)	−0.0043 (17)
C27	0.053 (2)	0.041 (2)	0.064 (2)	0.0024 (17)	−0.0003 (17)	−0.0046 (17)
C28	0.051 (2)	0.049 (2)	0.058 (2)	−0.0078 (17)	0.0013 (16)	−0.0068 (18)
C29	0.062 (2)	0.073 (3)	0.066 (2)	0.007 (2)	0.0058 (19)	−0.006 (2)
C30	0.080 (3)	0.089 (4)	0.082 (3)	0.010 (3)	0.016 (2)	−0.020 (3)
C31	0.093 (3)	0.101 (4)	0.063 (3)	−0.018 (3)	0.015 (2)	−0.013 (3)
C32	0.121 (4)	0.091 (4)	0.059 (3)	−0.009 (3)	−0.003 (3)	0.006 (3)
C33	0.092 (3)	0.058 (3)	0.068 (3)	0.005 (2)	−0.005 (2)	0.000 (2)
C34	0.0326 (17)	0.055 (2)	0.056 (2)	−0.0032 (16)	0.0046 (14)	0.0081 (17)
C35	0.0312 (17)	0.079 (3)	0.068 (2)	−0.0041 (18)	−0.0054 (15)	0.027 (2)
C36	0.062 (2)	0.046 (2)	0.0460 (19)	0.0012 (17)	−0.0010 (16)	0.0004 (16)
C37	0.057 (2)	0.044 (2)	0.0456 (18)	−0.0104 (16)	−0.0037 (15)	0.0069 (15)
C38	0.053 (2)	0.042 (2)	0.0464 (18)	−0.0059 (17)	−0.0050 (15)	0.0060 (16)
C39	0.0481 (19)	0.042 (2)	0.0445 (17)	0.0041 (16)	−0.0036 (14)	0.0043 (16)
C40	0.083 (3)	0.048 (2)	0.057 (2)	−0.004 (2)	−0.0036 (19)	−0.0060 (18)
C41	0.111 (4)	0.060 (3)	0.056 (2)	0.012 (3)	−0.008 (2)	−0.017 (2)
C42	0.084 (3)	0.087 (3)	0.050 (2)	0.018 (3)	0.007 (2)	−0.001 (2)
C43	0.072 (3)	0.094 (3)	0.058 (2)	−0.014 (2)	0.007 (2)	0.012 (2)
C44	0.064 (2)	0.065 (3)	0.050 (2)	−0.013 (2)	−0.0009 (17)	0.0007 (18)

### Geometric parameters (Å, °)

**Table:**

Cd1—N19	2.313 (3)	C9—C10	1.363 (7)
Cd1—N24^i^	2.322 (3)	C9—H9	0.9300
Cd1—N1	2.330 (3)	C10—C11	1.387 (6)
Cd1—N15	2.347 (3)	C10—H10	0.9300
Cd1—N4	2.351 (3)	C11—H11	0.9300
Cd1—N16	2.366 (3)	C12—H12	0.9300
Cd2—N13^ii^	2.308 (3)	C13—H13	0.9300
Cd2—N21	2.337 (3)	C14—C15	1.512 (4)
Cd2—N18	2.338 (3)	C14—H14A	0.9700
Cd2—N7	2.339 (3)	C14—H14B	0.9700
Cd2—N10	2.339 (3)	C15—C16	1.503 (5)
Cd2—N22	2.369 (3)	C15—H15A	0.9700
O1—C5	1.210 (4)	C15—H15B	0.9700
O2—C16	1.217 (4)	C16—C17	1.494 (5)
O3—C38	1.214 (4)	C17—C18	1.382 (5)
O4—C27	1.215 (4)	C17—C22	1.385 (5)
N1—C1	1.318 (4)	C18—C19	1.380 (5)
N1—C2	1.341 (4)	C18—H18	0.9300
N2—C2	1.306 (4)	C19—C20	1.363 (6)
N2—N3	1.350 (3)	C19—H19	0.9300
N3—C1	1.309 (4)	C20—C21	1.360 (6)
N3—C3	1.460 (4)	C20—H20	0.9300
N4—C13	1.315 (4)	C21—C22	1.386 (5)
N4—C12	1.350 (4)	C21—H21	0.9300
N5—C12	1.310 (4)	C22—H22	0.9300
N5—N6	1.360 (4)	C23—H23	0.9300
N6—C13	1.319 (4)	C24—H24	0.9300
N6—C14	1.469 (4)	C25—C26	1.511 (4)
N7—C24	1.323 (4)	C25—H25A	0.9700
N7—C23	1.348 (4)	C25—H25B	0.9700
N8—C23	1.308 (5)	C26—C27	1.509 (5)
N8—N9	1.362 (4)	C26—H26A	0.9700
N9—C24	1.319 (4)	C26—H26B	0.9700
N9—C25	1.458 (4)	C27—C28	1.490 (5)
N10—C34	1.315 (4)	C28—C33	1.382 (5)
N10—C35	1.345 (4)	C28—C29	1.383 (5)
N11—C35	1.307 (4)	C29—C30	1.384 (5)
N11—N12	1.357 (3)	C29—H29	0.9300
N12—C34	1.313 (4)	C30—C31	1.365 (6)
N12—C36	1.460 (4)	C30—H30	0.9300
N13—N14	1.165 (4)	C31—C32	1.366 (6)
N13—Cd2^i^	2.308 (3)	C31—H31	0.9300
N14—N15	1.158 (4)	C32—C33	1.379 (6)
N16—N17	1.165 (4)	C32—H32	0.9300
N17—N18	1.178 (4)	C33—H33	0.9300
N19—N20	1.162 (4)	C34—H34	0.9300
N20—N21	1.156 (4)	C35—H35	0.9300
N22—N23	1.163 (4)	C36—C37	1.510 (4)
N23—N24	1.180 (4)	C36—H36A	0.9700
N24—Cd1^ii^	2.322 (3)	C36—H36B	0.9700
C1—H1	0.9300	C37—C38	1.502 (4)
C2—H2	0.9300	C37—H37A	0.9700
C3—C4	1.510 (4)	C37—H37B	0.9700
C3—H3A	0.9700	C38—C39	1.492 (4)
C3—H3B	0.9700	C39—C44	1.383 (5)
C4—C5	1.505 (4)	C39—C40	1.384 (5)
C4—H4A	0.9700	C40—C41	1.382 (5)
C4—H4B	0.9700	C40—H40	0.9300
C5—C6	1.493 (4)	C41—C42	1.361 (6)
C6—C7	1.378 (5)	C41—H41	0.9300
C6—C11	1.379 (5)	C42—C43	1.366 (6)
C7—C8	1.384 (5)	C42—H42	0.9300
C7—H7	0.9300	C43—C44	1.386 (5)
C8—C9	1.354 (6)	C43—H43	0.9300
C8—H8	0.9300	C44—H44	0.9300
			
N19—Cd1—N24^i^	90.32 (12)	N4—C13—H13	124.7
N19—Cd1—N1	86.02 (12)	N6—C13—H13	124.7
N24^i^—Cd1—N1	172.10 (10)	N6—C14—C15	113.2 (3)
N19—Cd1—N15	97.65 (11)	N6—C14—H14A	108.9
N24^i^—Cd1—N15	89.15 (10)	C15—C14—H14A	108.9
N1—Cd1—N15	84.41 (10)	N6—C14—H14B	108.9
N19—Cd1—N4	174.39 (11)	C15—C14—H14B	108.9
N24^i^—Cd1—N4	94.54 (11)	H14A—C14—H14B	107.7
N1—Cd1—N4	89.49 (10)	C16—C15—C14	113.7 (3)
N15—Cd1—N4	85.23 (10)	C16—C15—H15A	108.8
N19—Cd1—N16	90.08 (10)	C14—C15—H15A	108.8
N24^i^—Cd1—N16	96.30 (11)	C16—C15—H15B	108.8
N1—Cd1—N16	90.71 (10)	C14—C15—H15B	108.8
N15—Cd1—N16	170.53 (11)	H15A—C15—H15B	107.7
N4—Cd1—N16	86.62 (9)	O2—C16—C17	120.9 (3)
N13^ii^—Cd2—N21	98.27 (11)	O2—C16—C15	119.7 (3)
N13^ii^—Cd2—N18	90.79 (12)	C17—C16—C15	119.4 (3)
N21—Cd2—N18	89.98 (10)	C18—C17—C22	118.8 (3)
N13^ii^—Cd2—N7	174.22 (10)	C18—C17—C16	118.8 (3)
N21—Cd2—N7	85.69 (10)	C22—C17—C16	122.4 (3)
N18—Cd2—N7	93.43 (10)	C19—C18—C17	120.7 (4)
N13^ii^—Cd2—N10	86.19 (11)	C19—C18—H18	119.6
N21—Cd2—N10	84.79 (10)	C17—C18—H18	119.6
N18—Cd2—N10	173.53 (10)	C20—C19—C18	119.8 (4)
N7—Cd2—N10	90.00 (10)	C20—C19—H19	120.1
N13^ii^—Cd2—N22	89.88 (10)	C18—C19—H19	120.1
N21—Cd2—N22	170.29 (11)	C21—C20—C19	120.4 (4)
N18—Cd2—N22	95.21 (11)	C21—C20—H20	119.8
N7—Cd2—N22	85.81 (9)	C19—C20—H20	119.8
N10—Cd2—N22	90.52 (10)	C20—C21—C22	120.5 (4)
C1—N1—C2	102.4 (3)	C20—C21—H21	119.8
C1—N1—Cd1	124.8 (2)	C22—C21—H21	119.8
C2—N1—Cd1	132.7 (2)	C17—C22—C21	119.8 (4)
C2—N2—N3	102.7 (3)	C17—C22—H22	120.1
C1—N3—N2	109.4 (3)	C21—C22—H22	120.1
C1—N3—C3	129.0 (3)	N8—C23—N7	114.7 (3)
N2—N3—C3	121.5 (3)	N8—C23—H23	122.6
C13—N4—C12	103.1 (3)	N7—C23—H23	122.6
C13—N4—Cd1	121.81 (19)	N9—C24—N7	110.9 (3)
C12—N4—Cd1	130.9 (2)	N9—C24—H24	124.6
C12—N5—N6	102.8 (3)	N7—C24—H24	124.6
C13—N6—N5	109.3 (3)	N9—C25—C26	114.5 (3)
C13—N6—C14	128.6 (3)	N9—C25—H25A	108.6
N5—N6—C14	121.8 (3)	C26—C25—H25A	108.6
C24—N7—C23	102.5 (3)	N9—C25—H25B	108.6
C24—N7—Cd2	120.30 (19)	C26—C25—H25B	108.6
C23—N7—Cd2	132.3 (2)	H25A—C25—H25B	107.6
C23—N8—N9	102.8 (3)	C27—C26—C25	112.7 (3)
C24—N9—N8	109.1 (3)	C27—C26—H26A	109.0
C24—N9—C25	128.3 (3)	C25—C26—H26A	109.0
N8—N9—C25	122.2 (3)	C27—C26—H26B	109.0
C34—N10—C35	102.2 (3)	C25—C26—H26B	109.0
C34—N10—Cd2	125.1 (2)	H26A—C26—H26B	107.8
C35—N10—Cd2	132.6 (2)	O4—C27—C28	121.2 (3)
C35—N11—N12	102.5 (3)	O4—C27—C26	119.7 (3)
C34—N12—N11	109.1 (3)	C28—C27—C26	119.1 (3)
C34—N12—C36	129.1 (3)	C33—C28—C29	118.7 (4)
N11—N12—C36	121.7 (3)	C33—C28—C27	118.6 (3)
N14—N13—Cd2^i^	124.6 (2)	C29—C28—C27	122.7 (3)
N15—N14—N13	175.5 (3)	C28—C29—C30	119.9 (4)
N14—N15—Cd1	131.1 (2)	C28—C29—H29	120.1
N17—N16—Cd1	122.6 (2)	C30—C29—H29	120.1
N16—N17—N18	178.1 (4)	C31—C30—C29	120.7 (4)
N17—N18—Cd2	115.8 (2)	C31—C30—H30	119.7
N20—N19—Cd1	126.8 (2)	C29—C30—H30	119.7
N21—N20—N19	175.7 (3)	C30—C31—C32	119.9 (4)
N20—N21—Cd2	129.5 (2)	C30—C31—H31	120.0
N23—N22—Cd2	124.2 (2)	C32—C31—H31	120.0
N22—N23—N24	178.4 (4)	C31—C32—C33	120.0 (5)
N23—N24—Cd1^ii^	116.1 (2)	C31—C32—H32	120.0
N3—C1—N1	110.9 (3)	C33—C32—H32	120.0
N3—C1—H1	124.6	C32—C33—C28	120.7 (4)
N1—C1—H1	124.6	C32—C33—H33	119.6
N2—C2—N1	114.6 (3)	C28—C33—H33	119.6
N2—C2—H2	122.7	N12—C34—N10	111.3 (3)
N1—C2—H2	122.7	N12—C34—H34	124.3
N3—C3—C4	112.9 (3)	N10—C34—H34	124.3
N3—C3—H3A	109.0	N11—C35—N10	114.9 (3)
C4—C3—H3A	109.0	N11—C35—H35	122.6
N3—C3—H3B	109.0	N10—C35—H35	122.6
C4—C3—H3B	109.0	N12—C36—C37	113.8 (3)
H3A—C3—H3B	107.8	N12—C36—H36A	108.8
C5—C4—C3	113.8 (3)	C37—C36—H36A	108.8
C5—C4—H4A	108.8	N12—C36—H36B	108.8
C3—C4—H4A	108.8	C37—C36—H36B	108.8
C5—C4—H4B	108.8	H36A—C36—H36B	107.7
C3—C4—H4B	108.8	C38—C37—C36	113.7 (3)
H4A—C4—H4B	107.7	C38—C37—H37A	108.8
O1—C5—C6	121.4 (3)	C36—C37—H37A	108.8
O1—C5—C4	120.2 (3)	C38—C37—H37B	108.8
C6—C5—C4	118.5 (3)	C36—C37—H37B	108.8
C7—C6—C11	118.7 (3)	H37A—C37—H37B	107.7
C7—C6—C5	122.5 (3)	O3—C38—C39	120.8 (3)
C11—C6—C5	118.8 (3)	O3—C38—C37	120.3 (3)
C6—C7—C8	120.2 (4)	C39—C38—C37	118.9 (3)
C6—C7—H7	119.9	C44—C39—C40	118.7 (3)
C8—C7—H7	119.9	C44—C39—C38	122.4 (3)
C9—C8—C7	120.2 (4)	C40—C39—C38	118.9 (3)
C9—C8—H8	119.9	C41—C40—C39	120.4 (4)
C7—C8—H8	119.9	C41—C40—H40	119.8
C8—C9—C10	120.7 (4)	C39—C40—H40	119.8
C8—C9—H9	119.6	C42—C41—C40	120.3 (4)
C10—C9—H9	119.6	C42—C41—H41	119.8
C9—C10—C11	119.5 (4)	C40—C41—H41	119.8
C9—C10—H10	120.3	C41—C42—C43	120.2 (4)
C11—C10—H10	120.3	C41—C42—H42	119.9
C6—C11—C10	120.6 (4)	C43—C42—H42	119.9
C6—C11—H11	119.7	C42—C43—C44	120.2 (4)
C10—C11—H11	119.7	C42—C43—H43	119.9
N5—C12—N4	114.2 (3)	C44—C43—H43	119.9
N5—C12—H12	122.9	C39—C44—C43	120.2 (4)
N4—C12—H12	122.9	C39—C44—H44	119.9
N4—C13—N6	110.7 (3)	C43—C44—H44	119.9
			
N19—Cd1—N1—C1	−110.3 (3)	C1—N1—C2—N2	0.3 (4)
N24^i^—Cd1—N1—C1	−47.7 (8)	Cd1—N1—C2—N2	−175.3 (3)
N15—Cd1—N1—C1	−12.2 (3)	C1—N3—C3—C4	120.8 (4)
N4—Cd1—N1—C1	73.0 (3)	N2—N3—C3—C4	−61.7 (4)
N16—Cd1—N1—C1	159.7 (3)	N3—C3—C4—C5	−72.4 (4)
N19—Cd1—N1—C2	64.4 (3)	C3—C4—C5—O1	4.5 (5)
N24^i^—Cd1—N1—C2	127.0 (7)	C3—C4—C5—C6	−174.6 (3)
N15—Cd1—N1—C2	162.5 (3)	O1—C5—C6—C7	−177.7 (4)
N4—Cd1—N1—C2	−112.3 (3)	C4—C5—C6—C7	1.4 (5)
N16—Cd1—N1—C2	−25.6 (3)	O1—C5—C6—C11	0.5 (5)
C2—N2—N3—C1	0.2 (4)	C4—C5—C6—C11	179.6 (3)
C2—N2—N3—C3	−177.7 (3)	C11—C6—C7—C8	0.3 (6)
N19—Cd1—N4—C13	15.4 (12)	C5—C6—C7—C8	178.5 (4)
N24^i^—Cd1—N4—C13	−134.7 (3)	C6—C7—C8—C9	−0.9 (7)
N1—Cd1—N4—C13	52.1 (3)	C7—C8—C9—C10	1.3 (8)
N15—Cd1—N4—C13	136.5 (3)	C8—C9—C10—C11	−1.1 (8)
N16—Cd1—N4—C13	−38.6 (3)	C7—C6—C11—C10	−0.1 (6)
N19—Cd1—N4—C12	−137.3 (10)	C5—C6—C11—C10	−178.3 (4)
N24^i^—Cd1—N4—C12	72.6 (3)	C9—C10—C11—C6	0.5 (7)
N1—Cd1—N4—C12	−100.6 (3)	N6—N5—C12—N4	−0.2 (4)
N15—Cd1—N4—C12	−16.2 (3)	C13—N4—C12—N5	−0.1 (4)
N16—Cd1—N4—C12	168.7 (3)	Cd1—N4—C12—N5	156.3 (3)
C12—N5—N6—C13	0.4 (4)	C12—N4—C13—N6	0.3 (4)
C12—N5—N6—C14	−173.4 (3)	Cd1—N4—C13—N6	−158.8 (2)
N13^ii^—Cd2—N7—C24	−2.9 (12)	N5—N6—C13—N4	−0.5 (4)
N21—Cd2—N7—C24	−136.3 (3)	C14—N6—C13—N4	172.7 (3)
N18—Cd2—N7—C24	133.9 (2)	C13—N6—C14—C15	126.3 (3)
N10—Cd2—N7—C24	−51.6 (2)	N5—N6—C14—C15	−61.2 (4)
N22—Cd2—N7—C24	39.0 (2)	N6—C14—C15—C16	−72.9 (4)
N13^ii^—Cd2—N7—C23	147.6 (9)	C14—C15—C16—O2	3.5 (5)
N21—Cd2—N7—C23	14.2 (3)	C14—C15—C16—C17	−176.7 (3)
N18—Cd2—N7—C23	−75.5 (3)	O2—C16—C17—C18	5.0 (5)
N10—Cd2—N7—C23	99.0 (3)	C15—C16—C17—C18	−174.8 (3)
N22—Cd2—N7—C23	−170.5 (3)	O2—C16—C17—C22	−174.8 (4)
C23—N8—N9—C24	−0.4 (4)	C15—C16—C17—C22	5.4 (5)
C23—N8—N9—C25	172.9 (3)	C22—C17—C18—C19	−0.7 (6)
N13^ii^—Cd2—N10—C34	112.7 (3)	C16—C17—C18—C19	179.5 (3)
N21—Cd2—N10—C34	14.0 (3)	C17—C18—C19—C20	0.5 (6)
N18—Cd2—N10—C34	50.3 (10)	C18—C19—C20—C21	0.4 (7)
N7—Cd2—N10—C34	−71.7 (3)	C19—C20—C21—C22	−1.1 (7)
N22—Cd2—N10—C34	−157.5 (3)	C18—C17—C22—C21	0.1 (6)
N13^ii^—Cd2—N10—C35	−63.6 (3)	C16—C17—C22—C21	179.9 (3)
N21—Cd2—N10—C35	−162.3 (3)	C20—C21—C22—C17	0.8 (6)
N18—Cd2—N10—C35	−126.0 (8)	N9—N8—C23—N7	0.2 (4)
N7—Cd2—N10—C35	112.0 (3)	C24—N7—C23—N8	0.1 (4)
N22—Cd2—N10—C35	26.2 (3)	Cd2—N7—C23—N8	−154.1 (3)
C35—N11—N12—C34	−0.2 (4)	N8—N9—C24—N7	0.5 (4)
C35—N11—N12—C36	177.0 (3)	C25—N9—C24—N7	−172.2 (3)
Cd2^i^—N13—N14—N15	173 (4)	C23—N7—C24—N9	−0.4 (4)
N13—N14—N15—Cd1	149 (4)	Cd2—N7—C24—N9	157.7 (2)
N19—Cd1—N15—N14	−90.0 (3)	C24—N9—C25—C26	−131.6 (3)
N24^i^—Cd1—N15—N14	0.2 (3)	N8—N9—C25—C26	56.5 (4)
N1—Cd1—N15—N14	−175.2 (3)	N9—C25—C26—C27	75.4 (4)
N4—Cd1—N15—N14	94.9 (3)	C25—C26—C27—O4	−8.1 (5)
N16—Cd1—N15—N14	125.5 (6)	C25—C26—C27—C28	170.8 (3)
N19—Cd1—N16—N17	−36.4 (3)	O4—C27—C28—C33	−5.6 (5)
N24^i^—Cd1—N16—N17	−126.7 (3)	C26—C27—C28—C33	175.5 (3)
N1—Cd1—N16—N17	49.6 (3)	O4—C27—C28—C29	172.6 (4)
N15—Cd1—N16—N17	108.5 (6)	C26—C27—C28—C29	−6.3 (5)
N4—Cd1—N16—N17	139.1 (3)	C33—C28—C29—C30	−0.1 (6)
Cd1—N16—N17—N18	−116 (12)	C27—C28—C29—C30	−178.3 (4)
N16—N17—N18—Cd2	−161 (11)	C28—C29—C30—C31	−0.2 (7)
N13^ii^—Cd2—N18—N17	−145.7 (3)	C29—C30—C31—C32	0.5 (7)
N21—Cd2—N18—N17	−47.4 (3)	C30—C31—C32—C33	−0.4 (8)
N7—Cd2—N18—N17	38.3 (3)	C31—C32—C33—C28	0.1 (7)
N10—Cd2—N18—N17	−83.5 (9)	C29—C28—C33—C32	0.2 (6)
N22—Cd2—N18—N17	124.4 (3)	C27—C28—C33—C32	178.5 (4)
N24^i^—Cd1—N19—N20	84.1 (4)	N11—N12—C34—N10	0.6 (4)
N1—Cd1—N19—N20	−103.0 (4)	C36—N12—C34—N10	−176.3 (3)
N15—Cd1—N19—N20	173.2 (3)	C35—N10—C34—N12	−0.7 (4)
N4—Cd1—N19—N20	−66.1 (12)	Cd2—N10—C34—N12	−177.9 (2)
N16—Cd1—N19—N20	−12.2 (4)	N12—N11—C35—N10	−0.3 (4)
Cd1—N19—N20—N21	179 (100)	C34—N10—C35—N11	0.6 (4)
N19—N20—N21—Cd2	−145 (5)	Cd2—N10—C35—N11	177.5 (2)
N13^ii^—Cd2—N21—N20	94.3 (3)	C34—N12—C36—C37	−124.4 (4)
N18—Cd2—N21—N20	3.5 (3)	N11—N12—C36—C37	59.1 (4)
N7—Cd2—N21—N20	−90.0 (3)	N12—C36—C37—C38	72.5 (4)
N10—Cd2—N21—N20	179.6 (3)	C36—C37—C38—O3	−5.9 (5)
N22—Cd2—N21—N20	−119.0 (6)	C36—C37—C38—C39	174.0 (3)
N13^ii^—Cd2—N22—N23	36.0 (3)	O3—C38—C39—C44	174.2 (4)
N21—Cd2—N22—N23	−111.2 (6)	C37—C38—C39—C44	−5.8 (5)
N18—Cd2—N22—N23	126.8 (3)	O3—C38—C39—C40	−4.6 (5)
N7—Cd2—N22—N23	−140.1 (3)	C37—C38—C39—C40	175.4 (3)
N10—Cd2—N22—N23	−50.2 (3)	C44—C39—C40—C41	0.4 (6)
Cd2—N22—N23—N24	115 (14)	C38—C39—C40—C41	179.2 (3)
N22—N23—N24—Cd1^ii^	162 (100)	C39—C40—C41—C42	−0.1 (6)
N2—N3—C1—N1	0.0 (4)	C40—C41—C42—C43	−0.3 (7)
C3—N3—C1—N1	177.7 (3)	C41—C42—C43—C44	0.4 (7)
C2—N1—C1—N3	−0.1 (4)	C40—C39—C44—C43	−0.3 (5)
Cd1—N1—C1—N3	175.9 (2)	C38—C39—C44—C43	−179.1 (3)
N3—N2—C2—N1	−0.3 (5)	C42—C43—C44—C39	−0.1 (6)

Symmetry codes: (i) *x*−1, *y*, *z*; (ii) *x*+1, *y*, *z*.
